# Supplementary figures and images for: Genetic mutational status of genes regulating epigenetics: Role of the histone methyltransferase KMT2D in triple negative breast tumors
Source: PLoS One. 2019 Apr 16;14(4):e0209134. doi: 10.1371/journal.pone.0209134 (PMC6467442; doi:10.1371/journal.pone.0209134)

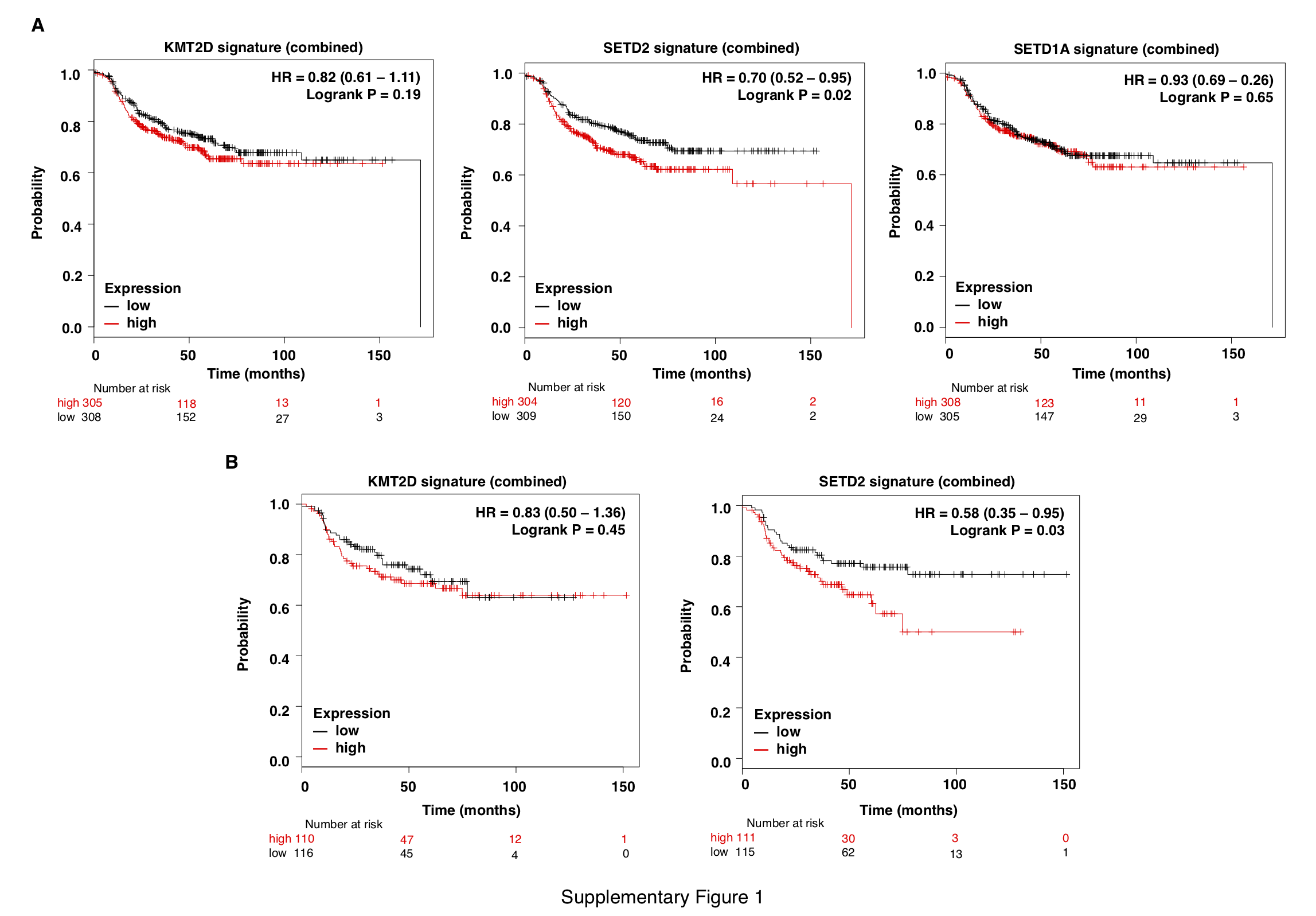

Supplement: S1 Fig — A. all breast cancer patients. B. Triple negative breast cancer patients. (TIFF) [file pone.0209134.s001.tiff]

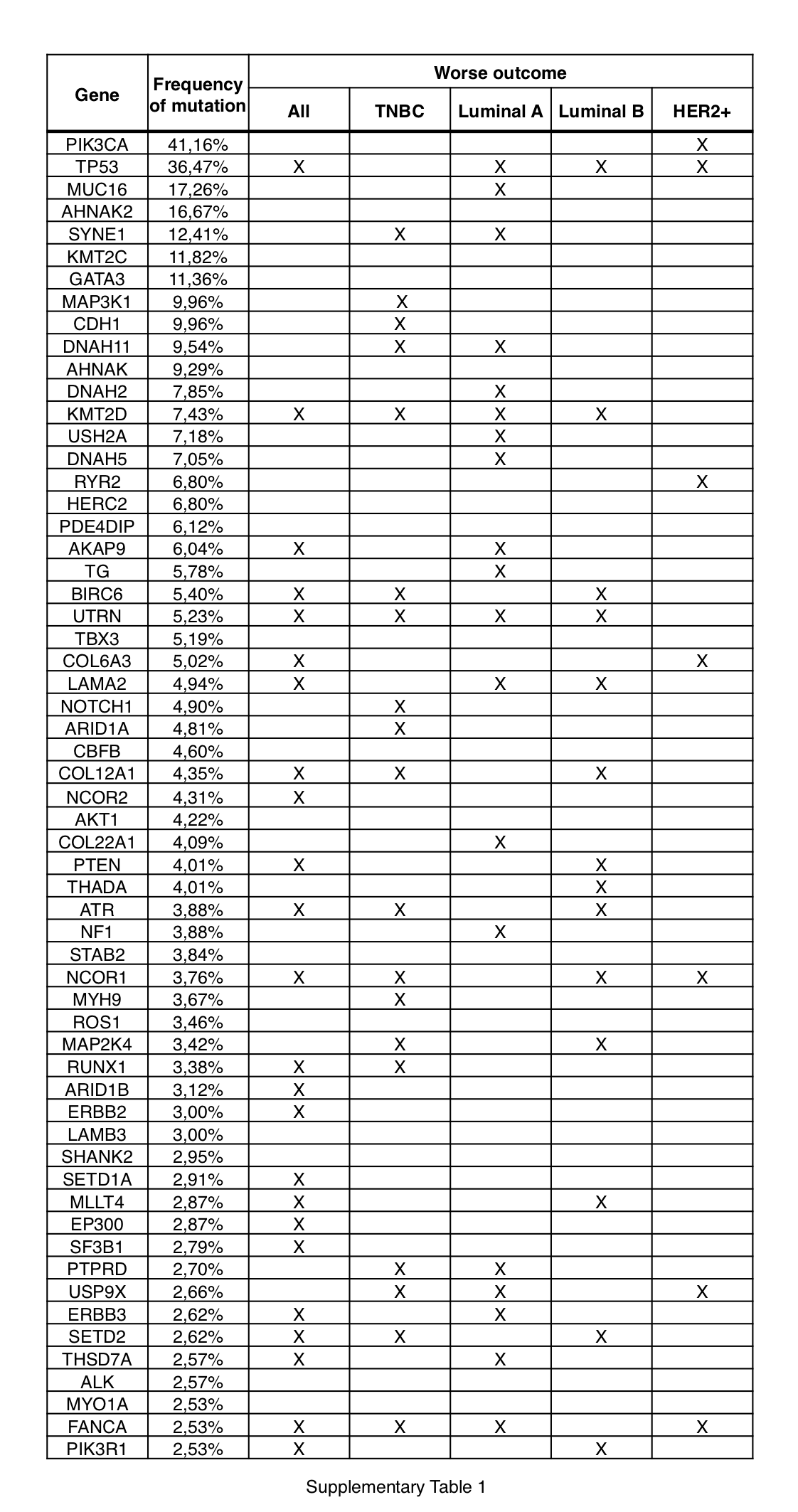

Supplement: S1 Table — Genes with worse outcome for each cancer subtype are shown in the table. (TIFF) [file pone.0209134.s002.tiff]

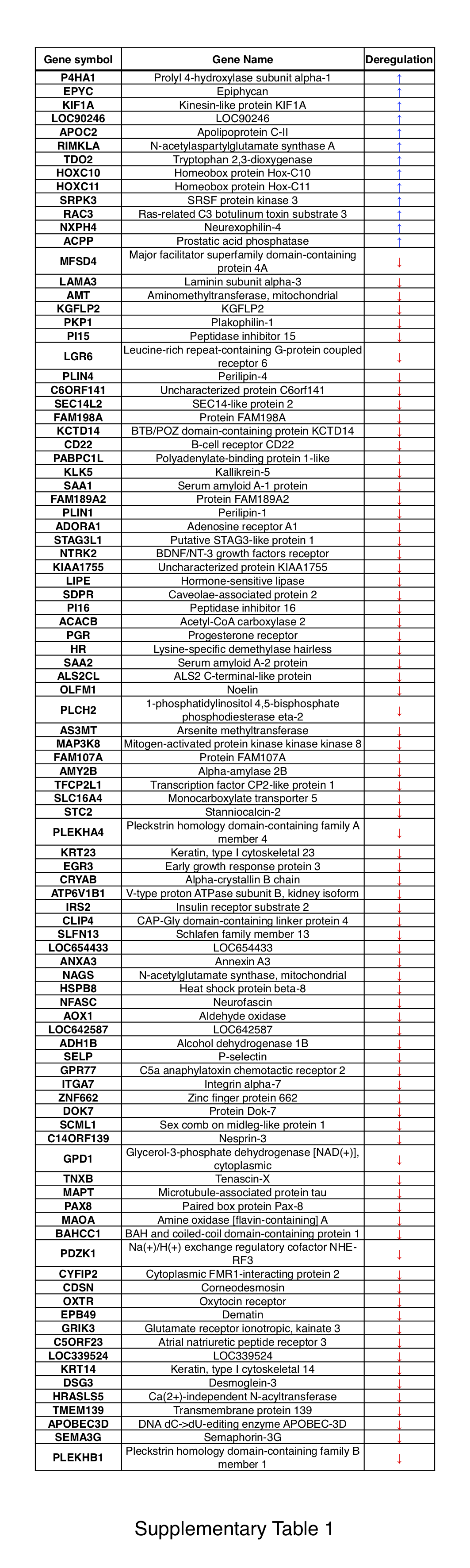

Supplement: S2 Table — Genes found to be upregulated or downregulated in the KMT2D mutational signature. Genotype-2-Outcome database was used for this analysis. (TIFF) [file pone.0209134.s003.tiff]

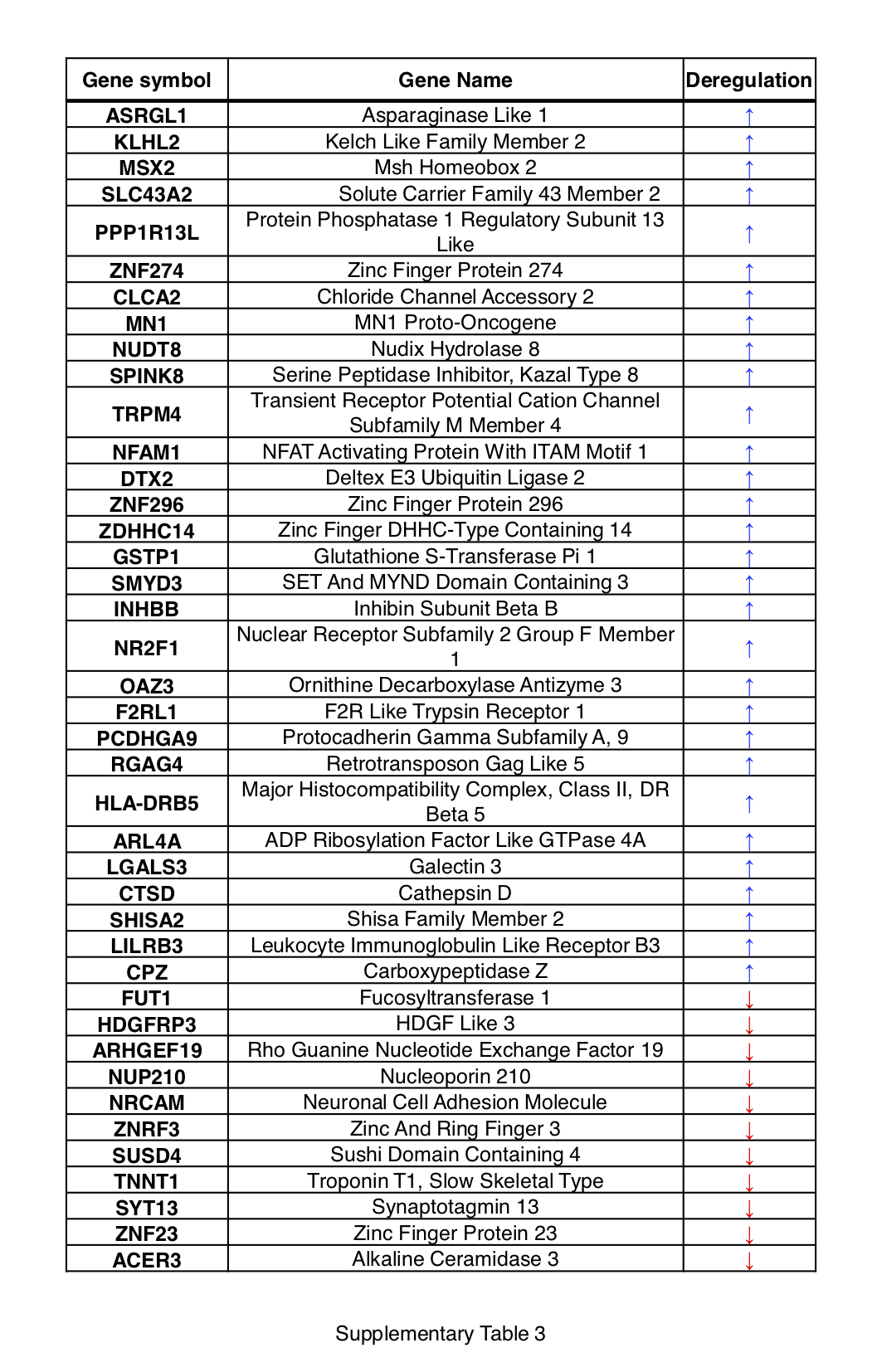

Supplement: S3 Table — Genes found to be upregulated or downregulated in the SETD1A mutational signature. Genotype-2-Outcome database was used for this analysis. (TIFF) [file pone.0209134.s004.tiff]

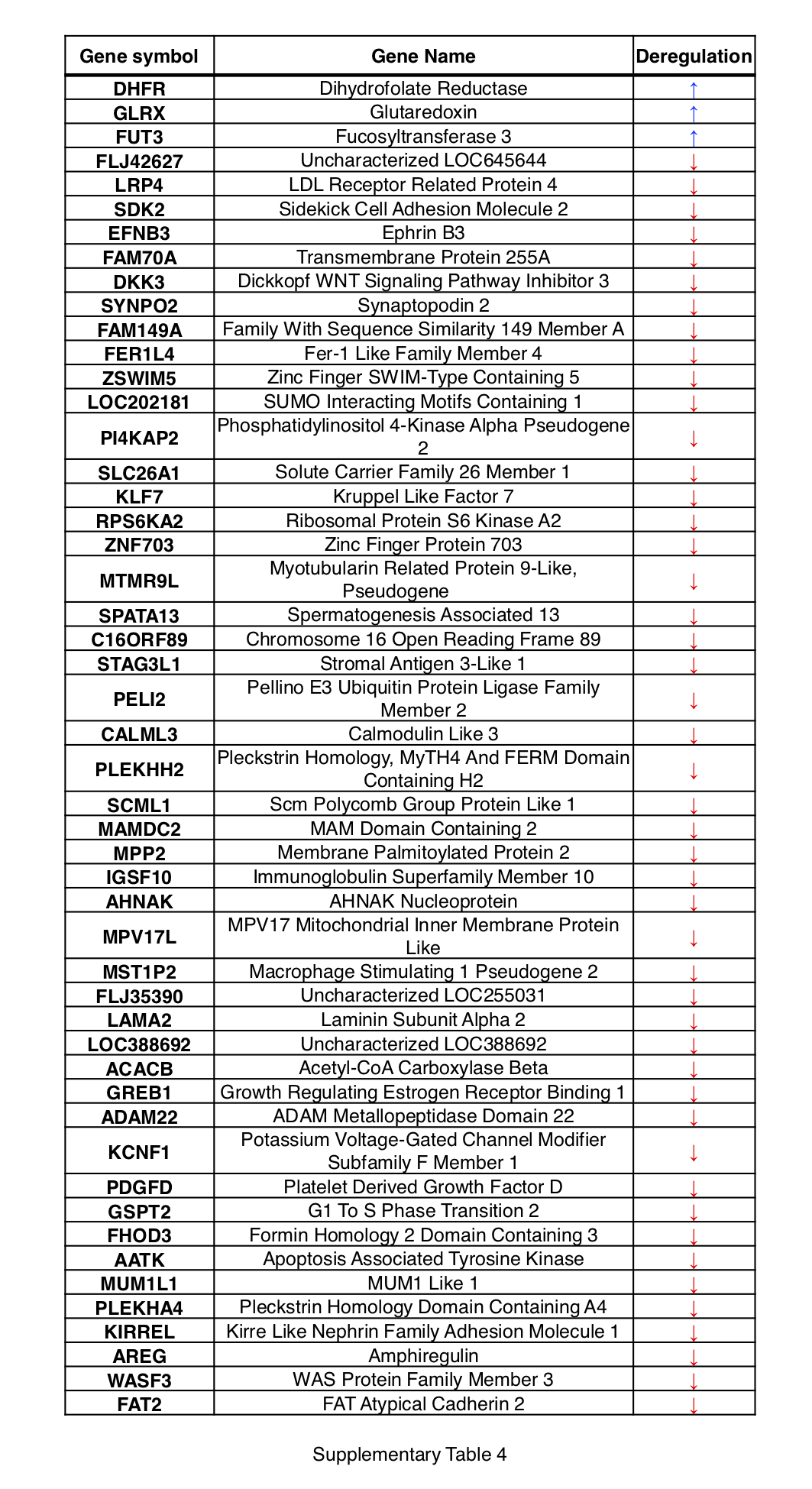

Supplement: S4 Table — Genes found to be upregulated or downregulated in the SETD2 mutational signature. Genotype-2-Outcome database was used for this analysis. (TIFF) [file pone.0209134.s005.tiff]
